# Incidence of Road Traffic Injury and Associated Factors among Patients Visiting the Emergency Department of Tikur Anbessa Specialized Teaching Hospital, Addis Ababa, Ethiopia

**DOI:** 10.1155/2014/439818

**Published:** 2014-08-07

**Authors:** Bewket Tadesse Tiruneh, Berihun Assefa Dachew, Berhanu Boru Bifftu

**Affiliations:** Department of Nursing, College of Medicine and Health Sciences, University of Gondar, P.O. Box 196, North Gondar Administrative Zone, Gondar, Ethiopia

## Abstract

*Background*. Road traffic injuries are a major public health issue. The problem is increasing in Africa. *Objective*. To assess the incidence of road traffic injury and associated factors among patients visiting the emergency department of Tikur Anbessa Specialized Teaching Hospital, Addis Ababa, Ethiopia. *Methods*. Institutional based cross-sectional study design was conducted. A total of 356 systematically selected study subjects were included in the study. Bivariate and multivariate logistic regressions were performed to identify associated factors with road traffic injury. Odds ratios with 95% confidence interval were computed to determine the level of significance. *Results*. The incidence of road traffic injury in the emergency department of Tikur Anbessa Specialized Teaching Hospital was 36.8%. Being a farmer (AOR = 3.3; 95% CI = 1.06–10.13), conflict with family members (AOR = 7.7; 95% CI = 3.49–8.84), financial problem (AOR = 9.91; 95% CI = 4.79–6.48), psychological problem (AOR = 17.58; 95% CI = 7.70–12.14), and alcohol use (AOR = 2.98; 95% CI = 1.61–5.27) were independently associated with road traffic injury. *Conclusion and Recommendation*. In this study the incidence of road traffic injury was high. Alcohol is one of the most significant factors associated with Road Traffic Injury. Thus urgent education on the effect of alcohol is recommended.

## 1. Background

World Health Organization defines road traffic injury (RTI) as a fatal or nonfatal injury incurred as a result of a collision on a public road involving at least one moving vehicle and pedestrians. It is a major but neglected public health challenge that requires concerted efforts for effective and sustainable prevention. Worldwide, an estimated 1.2 million people are killed in road crashes each year and as many as 50 million are injured. Nevertheless, the tragedy behind these figures attracts less mass media attention than other, less frequent types of tragedy [1].

Furthermore, according to the World Health Organization, the number of road traffic deaths is expected to increase by 80% up to 2020 [2]. Globally, road traffic injuries are ranked ninth among the leading causes of disability adjusted life years lost (DALY), and their ranking is projected to rise to third by 2020 [3, 4].

World report on road traffic injury showed that the number of road traffic injuries has continued to rise in the whole world, but there has been an overall downward trend in road traffic deaths in high-income countries since the 1970s and an increase in many of the low-income and middle-income countries. Deaths related to RTI are predicted to increase by 83% in low-income and middle-income countries and to decrease by 27% in high-income countries. 90% of road traffic deaths occurred in low-income and middle-income countries, where 81% of the world's population live and own about 20% of the world's vehicles [5].

African countries had the highest mortality rate, with 28.3 deaths per 100 000 populations [6]. The problem is increasing at a fast rate in African countries due to rapid motorization and other factors. While road traffic accidents account for about one-quarter of injury-related deaths in the continent overall, in Egypt 64%, in Tunisia 58%, and in Morocco 51% were injury-related deaths in 2008. In Libya 43%, in Djibouti 42%, in Mauritius 37%, in Namibia 36%, and in Niger 34% have road traffic accident related deaths. The most economically active people (aged 15–59) are at the greatest risk of dying as a result of RTI. For this age group, road traffic accidents affected more than three times as many males as females. Overall, 5% of deaths among males aged 15–59 are attributable to road traffic accidents, but this percentage rises to 6.5% for males in the 15–29 age group in Sub-Saharan Africa. Deaths due to road traffic accidents among males aged 15–59 far exceed those due to malaria, diabetes mellitus, and respiratory or digestive diseases [7].

Kenya has one of the highest road fatality rates in Africa at 68 deaths per 10 000 registered vehicles and between 45 and 60% of admissions to surgical wards in public hospitals as a result of road traffic injuries [8]. The Ethiopian National Road Safety Coordination Office cites a road crash fatality rate of 114 deaths per 10 000 vehicles per year [9]. Officially, 81% of crashes in Ethiopia are attributed to driver error. Driver impairment is rarely recorded as a contributory factor and government officials told us that they believe Khat (*Catha edulis*) use is a major cause of driver error and crashes [10].

The significance of this study is that effective road traffic injury (RTI) prevention, patient care, and rehabilitation all require a prior understanding of the incidence of RTI and associated factors in patients visiting the emergency department (ED) of health institutions. The results of this study will lead to an organized RTI data that provide good information about incidence of RTI and associated factors requiring ED visit and/or hospitalization. This will help to develop an effective legislation and educational response towards the problem.

This study was aimed at assessing the incidence of road traffic injury and associated factors among patients visiting the emergency department of Tikur Anbessa Specialized Teaching Hospital, Addis Ababa, Ethiopia.

## 2. Materials and Method

### 2.1. Study Design

Institutional based cross-sectional study was conducted at the Emergency Department of Tikur Anbessa Specialized Teaching Hospital, Addis Ababa, Ethiopia.

### 2.2. Study Area and Period

Ethiopia is Africa's second most populous country and its economy is largely depending on agriculture. There are air and land transportation services. Addis Ababa is the capital city of Ethiopia where Tikur Anbessa Specialized Teaching Hospital (TASTH) is located. This study was conducted in the emergency department of TASTH from April 30, 2013, to August 30, 2013. It is the largest of all hospitals in Ethiopia and provides a tertiary level referral treatment with a 24-hour emergency service.

### 2.3. Source and Study Population

All injured patients were in the Emergency Department of Tikur Anbessa Specialized Teaching Hospital during the study period.

#### 2.3.1. Inclusion Criteria

Patients who were visited at the Adult Emergency Department of Tikur Anbessa Specialized Teaching Hospital from April 30, 2013, to August 30, 2013 because of injury were included.

#### 2.3.2. Exclusion Criteria

Those injured cases that need immediate transfers to other hospitals because of organizational problem and/or lack of bed during the day of the data collection and victims of injury with repeated attendance were excluded.

### 2.4. Sampling Procedure

Sample size was calculated by using a single population proportion formula (*P* = 30.3%). Incidence of RTI [11] and a total of 356 study subjects were included in this study. We employed a systematic random sampling technique using the entry point to the triage seat of injured patients as sampling frame. Thus out of a total of 1354 injured patients b/n April 30, 2013, to August 30, 2013, 356 patients were selected using systematic random sampling technique.

### 2.5. Data Collection Tool and Procedure

In this study triage nurses collected data using a pretested questionnaire from the sample population according to the inclusion criteria. Data collection tool was adapted from injury surveillance guideline document of WHO developed in 2001. The data was collected by a face to face interview of the injured patients, informants, attendants, and families using the data collection instruments and there was also observation of the patient whether he/she took alcohol and his/her chart was reviewed for clinical data.

### 2.6. Data Quality Management

To maintain data quality, training was given for data collectors and supervisors for one day. Supervision was carried out on daily bases to check completeness and consistency both by the supervisors and by principal investigator. Correctly completed questioner was collected from data collectors.

### 2.7. Data Processing and Analysis

The collected data was entered and cleaned using EPI INFO and analyzed using SPSS for windows version 20 and frequency distribution and percentage calculation were made to describe sociodemographic characteristics. Percentages of admissions to hospital for treatment of RTI were calculated and it was examined by place, time, and other measures of descriptive statistics that were determined. Odds ratio with its corresponding 95% CI was determined to look into associations between variables. Binary logistic regression was used to check variables associated with the dependent variable. Those variables found to have *P* values of ≤0.2 were fitted to multivariable logistic regression to control the effects of confounders. Odds ratios with 95% CI were computed and variables having *P* values ≤0.05 in the multiple logistic regression models were considered significantly associated with the dependent variable. Model fitness was checked with the assumptions of Hosmer and Lemeshow goodness of a fit test (*P* = 0.7).

### 2.8. Ethical Considerations

A formal letter was secured after the research project was approved by Ethics and Research Committee at College of Health Science, Addis Ababa University, prior to commencement of the study. Written consent was obtained from each sample subjects to conduct the interview.

## 3. Result

### 3.1. Sociodemographic Characteristics of the Study Population

A total of 356 injury victims were consented and agreed to participate in this study. Males outnumbered females by a sex ratio of 3 : 0.7. More than 76% of these patients were found to be between the age of 20 and 59. The commonest occupations were farmer 74 (20.8%) and civil servant 72 (20.2). Majority of the participants (48.9%) were single and most of the injury victims (43.5%) had primary education (Table 1).

### 3.2. Incidence of the Road Traffic Injury

In this study, the incidence of road traffic injuries was 36.8%. The majority of these victims were pedestrians which accounts for 94 people (71.7%), followed by passengers which consist of 17 people (13%) and drivers which constitute 16 people (12.2%), and the rest were assistants of the drivers. People living in urban areas accounted for 74% of the road traffic injury. Most of these road traffic injuries occurred on the street and they account for 117 (89.3%) of the injuries followed by working area 13 injuries (10%) as well as 1 (0.8%) of the injury was occurred around the home surrounding. Seventy point two percent of the accidents occurred during the morning and the evening time followed by the afternoon time (25.9%). The rest happened during the midnight time. The majority of RTI cases 46 (35.1%) used minibus as a mode of transportation to come to the ED of the hospital after the event of the injury (Table 2).

The most frequent locations of the injury were head, neck, and face; 57 (43.5%) of patients had injuries in these areas. On top of that 34 (25.9%) and 17 (12.9%) of the patients had injuries involving the lower extremity and the upper limbs, respectively. For the reaming RTI victims, the injury involves the pelvic, the chest, and the spinal cord in 15 (11.4%), 6 (4.5%) and 2 (1.5%) of the cases correspondingly. Moderate injury requiring some skilled treatment was the leading outcome of the injuries constituting 97 (74.4%) of the injuries followed by severe injury that requires intensive surgical management, 20 (15%) (Table 3). The majority of the cases were admitted, 81 (61.8%), both in the surgical and in the orthopaedic wards and the remaining injuries, that is, 50 (38.16%) of the injuries, were treated and discharged.

### 3.3. Factors Associated with Road Traffic Injury

In the multivariate analysis selected socioeconomic factors, farmer, and conflict with family members, psychological problem, financial problem, and alcohol use were a significant risk factor for the occurrence of the RTI more prevalently in a specific group of the study population. Being a farmer was 3.4 times more likely to have RTI as compared to a trader (*P* = 0.04, AOR: 3.37, 95% CI: (1.0–10.13)), individuals who have conflict with family members were 7.7 times more likely to be injured in an RTI than those who do not have the conflict (*P* < 0.001, AOR: 7.6, 95% CI: (3.49–8.84)), and those individuals who have psychological problem were 17.6 times more likely to be injured in an RTI than individuals who do not have psychological problems (*P* < 0.001, AOR: 17.5, 95% CI: (7.70–12.14)). Moreover, in the analysis, it is also found that study subjects who have a financial problem were 9.9 times more likely being injured in an RTI as compared to those who do not have the problem (*P* < 0.001, AOR: 9.9, 95% CI: (4.7–6.48)) and those who drank alcohol were more likely to be injured in an RTI as compared to those who do not drink alcohol (*P* < 0.001, AOR, 2.9 (1.6–5.2)) (Table 4).

## 4. Discussion

This study revealed that incidence of road traffic injury was found to be 36.8%. This is relatively similar with the 35.2% in India [12], 27.7% in Kenya [13], 43.9% in Tanzania [14], 30.3% in Jimma University hospital l [11], and 33.8% in Addis Ababa [15]. This could be due to similarity of the study setting. Similarly the Indian study is also done in the same setting of medical institute in the capital city of the country where the vehicle population is high and this paper is almost in agreement with the Jimma University Hospital findings. The slit difference may come from the fact that Black Lion Hospital is the final destination of cases including RTI from all parts of Ethiopia. Thus this may explain the existence of this difference. Nevertheless, this study is not in line with the finding of the Nigerian study because the issue in Nigeria is documented as 90.6% [16]. This may result in the differences of the study period b/n the two studies.

In this study the RTI cases were predominantly male (78.7%). Again, it is possible to see how the study in India (88.22%) [12], Libya (81.5%) [17], and Kenyan (75%) [18] agrees with this finding. Moreover, this paper examines 41.6% of the RTI victims who were found to be between the ages of 20 and 29 followed by 30–39 (18.3%) years of age. This is in line with other similar studies done in Kenya [18].

People in the age group of 30–34 (33.2%) were the most affected group followed by 15–29 (23.4%) and in Tanzania the 15–44 years of age were the most affected age group in this form of injury (RTI) [19]. This may be explained by the fact that a large proportion of the developing countries population is young in age. This reminds us that the most productive segment of the population is highly affected by road traffic injury.

This study finds that most of the road traffic injuries occurred during the morning and the evening time equally 35.11% followed by the afternoon time 25.95%. The Indian [12] study also makes this point similar in that the peak time for fatal accidents (7.26%) was between 10 PM and 11 PM followed by 9 PM to 10 PM (6.01%) and 53.20% of fatal accident occurring between 6 PM and 6 AM. This could be due to the fact that the transportation services in large cities continue up to the indicated time with high probability of alcohol consumption from the pedestrian side.

The findings of this work argue that most RTI incidence occurred among the pedestrian 94 (26.4%) followed by passenger 18 (5.1%), driver 16 (4.5%), and assistant of the driver 4 (1.1%); additionally 55.31% of the pedestrian, 93.75 of the driver, 66.6% of the passenger, and 75% of the assistant of the driver were admitted or referred to other hospitals for admission. Likewise, Al-Jalaa hospital [17] study makes this case that pedestrian, passenger, and driver were the most victims of RTI. Conversely, this study argues on the view that drivers constitute 28.3% of the RTI patients admitted to the hospital and passengers formed 37.7% of the admission. This may be explained by the fact that the road environment in Ethiopia is known to be relatively unsafe and uncomfortable compared to that of higher income countries and the country has a mix of road users. Thus this can increase the severity of injury that necessitates admission in the Emergency Department of Tikur Anbessa Specialized Teaching Hospital.

The findings of this paper also revealed that moderate injury requiring some skilled treatment was the leading outcome of injury, 250 (70.2%), followed by severe injury, requiring intensive surgical management, 55 (15.4%), and minor injuries 36 (10.1%). On the other hand the study which was done in Jimma University Hospital [11] makes the case that 360 (32.7%) were classified as having severe injury, 455 (41.3%) as moderate injury, and 287 (26.0%) as minor injury. The contradiction between the two may be due to the fact that those severely injured cases in Addis Ababa may be taken into other nearby hospitals in the city immediately and it is also possible to say that severely injured cases have a limited chance of reaching the ED of TASTH as a result of the distance from other reigns of the country as compared to those moderately injured cases.

Our study also showed that those who drank alcohol were more likely to have road traffic injury as compared to those who do not use alcohol (*P* < 0.001, AOR: 2.918 (1.614–5.276)). Similarly it is possible to see how the Tirana study agrees with this one in that alcohol consumption (AOR, 5.09; 95% CI, 3.07–8.45) was significantly associated with RTI [20]. In our study significantly more farmers had RTI (AOR = 3.37; 95% CI = 1.06–10.13) than traders and those victims who have a conflict with family members had more RTI as compared to those who do not have the conflict (AOR = 7.7; 95% CI = 3.49–8.84). This study finds out that there is no association between road traffic injury and sex. However, fewer males had road traffic accident (AOR = 0.7, 95% CI = 0.5, 0.9) than females in Jimma University Specialized Hospital [11]. Moreover, a study on factors associated with severity of road traffic injuries, Thika, Kenya, shows that, in multivariate logistical regression road users, night time crashes remained independent risk factors for RTI [8, 16]. However, our study is not in the same position with such findings. This may be due to difference of method and time of investigation b/n the two studies.

This study has the following limitations. The methodology is cross-sectional so that chicken and egg dilemma still exists. The sample size is somehow small. The study focused on the host factors only. Moreover, injured cases may not come to the hospital. Thus, the overall injury incidence might have been underestimated.

## 5. Conclusion

This study has shown the incidence of road traffic injury in the emergency department of Tikur Anbessa Specialized Teaching Hospital as 36.8% which is high. Being a farmer, conflict with family members, psychological and financial problem, and alcohol drinking were found to be significantly associated with road traffic injury. Hence, suitable defensive strategy should be designed and implemented against RTI.

## Figures and Tables

**Table 1:** Sociodemographic characteristics of injury cases in the Emergency Department of Tikure Anbessa Specialized Teaching Hospital, Addis Ababa, Ethiopia, 2013 (*N* = 356).

Characteristics	Number	%
Sex		
Male	280	78.7
Female	76	21.3
Age		
<20	58	16.3
20–29	148	41.6
30–39	65	18.3
40–49	35	9.8
50–59	23	6.5
>60	27	7.6
Ethnicity		
Oromo	134	37.6
SNNP	75	21.1
Amhara	124	34.8
Tigrie	19	5.3
Others	4	1.1
Marital status		
Single	74	48.9
Married	47	41.3
Separated	23	6.5
Widowed	12	3.4
Educational status		
Illiterate	82	23.0
Read and write	4	3.9
Primary (1–8)	155	43.5
Secondary (9–12)	80	22.5
College diploma	2	.6
College degree	23	6.5
Income		
151–650	45	12.6
651–1400	44	12.4
1441–2350	24	6.7
2351–3550	7	2.0
3551–5000	6	1.7
No income	101	28.4
Unknown	129	36.2
Occupation		
Trader	36	10.1
Farmer	74	20.8
Civil servant	72	20.2
Student	53	14.9
Construction Worker	24	6.7
Day labourer	45	12.6
Unemployed	52	14.6
Residence		
Urban	244	68.5
Rural	112	31.5

**Table 2:** Mode of transportation used by road traffic injury victims in the Emergency Department of Tikur Anbessa Specialized Teaching Hospital, Addis Ababa, Ethiopia, 2013 (*N* = 131).

Mode	Number	Percentage
Minibus	46	35.1
Ambulance	30	23
Taxi	27	20.6
Private care	17	13
Bus	5	3.8
Heavy truck	2	1.5
Walking	2	1.5
Caring by people	1	0.7
Police transportation	1	0.7

Total	131	100

**Table 3:** Distribution of road traffic injury victims by severity of injury in the Emergency Department of Tikur Anbessa Specialized Teaching Hospital, Addis Ababa, Ethiopia (*N* = 131).

Severity of the injury	Number	Percentage
No apparent injury	4	3.0
Minor or superficial (e.g., bruises, minor cuts)	10	7.6
Moderate, requiring some skilled treatment (e.g., fractures, sutures)	97	74.4
Severe, requiring intensive medical/surgical management (e.g., internal hemorrhage)	20	15

Total	131	100

**Table 4:** Result of multivariate analysis for selected behavioural and environmental factors related to road traffic injury (RTI).

Variable	RTI		Crude odds ratio (COR)	Adjusted odds ratio (AOR)	*P* value
	Yes	No			
Occupation					
Farmer	14	60	3.835 (1.59–9.20)	3.37 (1.06–10.13)∗	0.04
Civil servant	37	35	.846 (.38–1.88)	.846 (.380–1.886)	
Student	17	36	1.895 (.79–4.53)	1.192 (.339–4.191)	
Construction worker	7	17	2.173 (.72–6.50)	1.192 (.339–4.191)	
Day laborer	18	27	1.342 (.55–3.25)	1.245 (.340–4.553)	
Unemployed	21	31	1.321 (.56–3.11)	.911 (.260–3.190)	
Trader	17	19	1	1	
Conflict with family members					
Yes	28	21	2.641 (1.43–4.88)	7.7 (3.49–8.84)∗	<0.001
No	103	204	1	1	
Psychological problem					
Yes	38	14	6.158 (1.38–11.90)	17.585 (7.70–12.14)∗	<0.001
No	93	211	1	1	
Financial problem					
Yes	40	27	3.223 (1.86 –5.57)	9.915 (4.79–6.48)∗	<0.001
No	91	198	1	1	
Alcohol use					
Yes	64	52	3.178 (2.00–5.04)	2.918 (1.61–5.27)∗	<0.001
No	67	173	**1**	**1**	

∗Significantly associated with RTI.
